# New approach for genomic characterisation of equine sarcoid-derived BPV-1/-2 using nanopore-based sequencing

**DOI:** 10.1186/s12985-021-01735-5

**Published:** 2022-01-06

**Authors:** Lien Gysens, Bert Vanmechelen, Maarten Haspeslagh, Piet Maes, Ann Martens

**Affiliations:** 1 Department of Surgery and Anaesthesiology of Domestic Animals, Faculty of Veterinary Medicine, Ghent University, Salisburylaan 133, 9820 Merelbeke, Belgium; 2 Laboratory of Clinical and Epidemiological Virology, Department of Microbiology, Immunology and Transplantation, Rega Institute for Medical Research, KU Leuven – University of Leuven, Herestraat 49/Box 1040, 3000 Leuven, Belgium

**Keywords:** Bovine papillomavirus, Equine sarcoid, Horse, Nanopore sequencing, Phylogenetic analysis

## Abstract

**Background:**

Bovine papillomavirus (BPV) types 1 and 2 play a central role in the etiology of the most common neoplasm in horses, the equine sarcoid. The unknown mechanism behind the unique variety in clinical presentation on the one hand and the host dependent clinical outcome of BPV-1 infection on the other hand indicate the involvement of additional factors. Earlier studies have reported the potential functional significance of intratypic sequence variants, along with the existence of sarcoid-sourced BPV variants. Therefore, intratypic sequence variation seems to be an important emerging viral factor. This study aimed to give a broad insight in sarcoid-sourced BPV variation and explore its potential association with disease presentation.

**Methods:**

In order to do this, a nanopore sequencing approach was successfully optimized for screening a wide spectrum of clinical samples. Specimens of each tumour were initially screened for BPV-1/-2 by quantitative real-time PCR. A custom-designed primer set was used on BPV-positive samples to amplify the complete viral genome in two multiplex PCR reactions, resulting in a set of overlapping amplicons. For phylogenetic analysis, separate alignments were made of all available complete genome sequences for BPV-1/-2. The resulting alignments were used to infer Bayesian phylogenetic trees.

**Results:**

We found substantial genetic variation among sarcoid-derived BPV-1, although this variation could not be linked to disease severity. Several of the BPV-1 genomes had multiple major deletions. Remarkably, the majority of them cluster within the region coding for late viral genes. Together with the extensiveness (up to 603 nucleotides) of the described deletions, this suggests an altered function of L1/L2 in disease pathogenesis.

**Conclusions:**

By generating a significant amount of complete-length BPV genomes, we succeeded to introduce next-generation sequencing into veterinary research focusing on the equine sarcoid, thus facilitating the first report of both nanopore-based sequencing of complete sarcoid-sourced BPV-1/-2 and the simultaneous nanopore sequencing of multiple complete genomes originating from a single clinical sample.

**Supplementary Information:**

The online version contains supplementary material available at 10.1186/s12985-021-01735-5.

## Background

Representing up to 90% of all dermatological tumours to affect horses, the equine sarcoid is the most commonly found skin tumour of horses and donkeys worldwide [1, 2]. Sarcoids are non-metastasizing, but persistent tumours of fibroblastic origin with a wide range of clinical entities, often occurring simultaneously within the same individual [3]. According to their gross morphology, five sarcoid types are described: occult, nodular, verrucous, fibroblastic and mixed, with the latter being a combination of several of these types [4, 5]. The reason for this unique variety in clinical presentation remains to be elucidated. As more advanced fibroblastic tumours have a less favorable prognosis, it is important to gain more insight into the origin and development of equine sarcoids.

Papillomaviruses are slowly evolving double-stranded DNA viruses, having an estimated substitution rate ranging between 2 × 10^−8^ and 5 × 10^−9^ substitutions per site per year [6], known to have species-specific biological characteristics [7]. They are ubiquitous in a wide range of vertebrate host species, often causing benign papillomas for which subsequent spontaneous regression is observed [8]. Members of the family *Papillomaviridae* typically share a similar genome organization, characterized by a double-stranded DNA genome of approximately 8 kbp containing a non-coding long control region (LCR) and eight open reading frames (ORFs). These ORFs encode six early proteins (E1–E2, E4–E7) and two late proteins (L1, L2). The transcription of early genes is responsible for episomal genome maintenance, regulation of cell growth and cell transformation [9–11]. Some RNA molecules containing late transcripts have been demonstrated within sarcoids [12, 13], yet it remains unclear whether this culminates in the production of infectious virions.

Uniquely, equine sarcoids are the result of natural cross-species infection by BPV-1, BPV-2 or BPV-13, classified in the genus *Deltapapillomavirus*. Co-infection with BPV-1 and BPV-2 in the same lesion has been observed [14], but is rather exceptional. The association between these genotypes and the etiology of equine sarcoids is well documented, although the mechanism of cross-species infection and the host-dependent clinical outcome of BPV infection, i.e. regressive fibropapillomas in cattle compared to equine sarcoids in horses, has not been elucidated. Since the earliest report of an equine sarcoid in 1936 [15], many studies have demonstrated the presence of BPV DNA, mRNA and proteins in virtually all equine sarcoids, making BPV the main extrinsic factor responsible for the development of sarcoid lesions [16–20]. Unlike the situation in the natural bovine host, a largely unknown mechanism results in sarcoid growth characterized by persistency and frequent recurrence following treatment [21].

The identification of a set of intra-type sequence variants in selected regions of sarcoid-sourced BPV has fueled suspicions of the existence of equine-adapted viral subspecies that might be favored within the equine host [20, 22, 23]. Moreover, the description of sarcoid dissemination within isolated populations [24, 25], further supports this hypothesis and suggests that these subspecies could be maintained within the equine population.

Collected data from previous literature indicate that sequence changes could affect the expression and function of viral proteins. This applies to both HPV-16 E6 proteins [26] and sequence variants in the LCR and the E2-ORF of BPV-1 isolated from equine sarcoids [27]. The functional significance of sequence variation suggests that BPV variation could alter biological properties and potentially represent an additional risk factor for more aggressive clinical behavior. In this context, the main objective of this report was to introduce nanopore sequencing technology to sarcoid research in order to provide an extensive full-length genomic characterisation of sarcoid-derived BPV-1/-2. For selected regions, like E5 and LCR, targeted in earlier reports, we sought to address the presence of mutations previously described as ‘potentially sarcoid-associated’ by other authors. However, as it is not known in which genomic fragments functionally significant mutations occur, we also aimed to identify new mutations in unexpected genetic regions. Therefore, we optimized a third-generation nanopore sequencing approach, which allows the simultaneous whole genome sequencing of multiple sequence variants originating from a single clinical sample. The sample set includes specimens of all different clinical types to elucidate if sequence variants contribute to the unique clinical presentation of the equine sarcoid. This would be analogous with the findings of Kurvinen et al. [28], who described an association between HPV intratypic variants and increased aggressive clinical behavior.

## Methods

### Sample set

This study includes samples of equine sarcoids and bovine papillomas originating from animals residing at different locations in Belgium and neighboring countries (France and Luxembourg). A variety of different sample types were included in this study: superficial tumour swabs, fine-needle aspirates (FNA), tumour tissue and paraffin-embedded tissue. Based on clinical evaluation of equine skin tumours, the subjects were divided into four groups in such a way that each sarcoid type was represented by a minimum set of 12 tumours: occult (n = 17), nodular (n = 18), verrucous (n = 15), fibroblastic (n = 12). Each tumour was identified and classified, based on their gross morphology according to the criteria of Pascoe and Knottenbelt [29]. In total, 62 equine sarcoids and four papillomas were included, originating from 36 horses and four bovids, respectively. Detailed information on each sample is provided in the online supplementary material (Additional file 1).

### BPV identification and typing

Specimens of each tumour were initially screened for BPV-1/-2 by quantitative real-time PCR [30]. Nucleic acids were extracted using the DNeasy Blood and Tissue Extraction Kit (Qiagen, Valencia, CA) according to the manufacturer’s instructions. Elution was done in a volume of 50 µl except for tissue samples, which were eluted in a volume of 100 µl. Prior to DNA-extraction, paraffin-embedded tissue was deparaffinized with three xylene and two 95% ethanol washes. For each sample, purified total DNA was analyzed along with its 1:10 dilution. Each qPCR run included positive controls and no-template controls (NTC). Positive controls constituted a quantified dilution series of pooled bovine papillomaviral DNA (type 1 and 2), obtained from multiple equine sarcoids. An oligonucleotide primer set (**f**-AATCGGGTGAGCAACCTTT, **r**-TGCTGTCTCCATCCTCTTCA) complementary to a consensus BPV sequence of the E1 ORF was used. BPV type-specific Double-Dye probes allowed differentiation between BPV-1 (**FAM**-CGTCAATCAGGTCTAAACGCCC-**BHQ1**) and BPV-2 (**Texas Red**-TCAACCAGGTCTAAGCGCCC-**BHQ2**). Reactions were performed in a 15 µl reaction volume on an iCycler iQ Real-Time PCR Detection System (Bio-Rad) using the iQ Supermix (Bio-Rad). The initial denaturation and *Taq* DNA polymerase activation was performed at 95 °C for 3 min, followed by forty-five cycles of denaturation at 95 °C for 20 s, allowing combined primer annealing and extension, at 60 °C for 40 s, during which fluorescence was measured. C_t_-values (threshold cycle) were obtained when the fluorescent signal was detected above the background in the exponential phase of amplification.

### BPV whole-genome sequencing

A custom-designed primer set was used to amplify the complete BPV genome in two multiplex PCR reactions, resulting in a set of overlapping amplicons. Initially, ten amplicons were used but this number was reduced to eight in later versions of the protocol. A complete overview of all used primers can be found in Additional file 2. Primers for all even and uneven amplicon numbers were pooled separately and primer pools were diluted to 10 µM. PCR was done using the Q5 Hot Start High-Fidelity 2X Master Mix (New England Biolabs, Ipswich, MA, USA), using 40 pmol primer pool 1 or 2 and 2.5 µl sample in a total reaction volume of 25 µl. PCR cycling was done using the following program: 30 s hot start at 98 °C, followed by 35 cycles of 15 s denaturation at 98 °C and 5 min extension at 65 °C. Amplicons were cleaned up by mixing both pools and using 1X Ampure XP beads (Beckman Coulter, Brea, CA, USA). Two wash steps with 70% ethanol were used prior to elution in 50 µl nuclease-free water. Quantification was done using the Qubit 1X dsDNA HS Assay Kit (Thermo Fisher Scientific, Waltham, MA, USA), after which 50 ng of each sample was used to prepare a barcoded nanopore sequencing library with the SQK-LSK109 kit and EXP-NBD104/EXP-NBD114 expansion packs.

### Data analysis

Nanopore fast5-files were basecalled and demultiplexed using Guppy v4.0.11 (Oxford Nanopore Technologies, Oxford, UK). Primer sequences were trimmed off using Porechop v0.2.4 and lower quality reads were removed using Filtlong v0.2.0. Minimap2 was used to map the remaining reads to the BPV-1/-2 RefSeq sequence, after which Medaka v1.0.1 (medaka_variant) was used to identify variants present in each sample and deduce consensus sequences.

### Phylogenetic analysis

MAFFT v7.310 was used to make alignments of all available complete genome sequences for BPV-1/-2. Separate alignments were made for BPV-1 and BPV-2. The resulting alignments were used to infer Bayesian phylogenetic trees using BEAST v1.10.4, following model selection using IQ-TREE. TN+I (BPV-1) and HKY+I (BPV-2) were selected as the models for nucleotide substitution and TreeAnnotator v1.10.4 was used to summarize maximum clade credibility trees once Markov chain Monte Carlo analyses had run sufficiently long (ESS > 200). FigTree v1.4.3 was used to visualize the resulting trees.

## Results

### Optimization of the nanopore sequencing protocol

To optimize our protocol, we performed amplification and nanopore sequencing on three sample batches (Additional file 1). The primer set used for the amplification of complete BPV genomes in batch 1 (V1) consisted of ten overlapping amplicons, divided across two pools, that spanned the complete BPV genome. These primers were degenerated at multiple positions (Additional file 2) to allow them to amplify nearly all members of the species *Deltapapillomavirus 4*, including types 1, 2 and 13. Comparable DNA amplification was observed in all twelve samples, but only for eleven samples, at least one complete genome sequence could be recovered from the nanopore data. Genomes were only considered complete if a minimum coverage threshold of 100x at each position was met, to ensure the absence of sequencing errors. The sample that failed to produce a complete genome had a low viral load, indicating a possible link with input sample quality. Interestingly, in one sample, two complete genomes could be retrieved from the data. In a second case, two variants of the same strain could be identified, characterized by the presence or absence of a 603-nt deletion. Additionally, the presence of additional partial genome fragments, corresponding to one or more amplicons, was noted in several samples, including the one sample that failed to yield a complete genome. One sample initially yielded a complete BPV-1 and BPV-2 genome, but closer inspection revealed that the BPV-1 genome was a chimaera of two BPV-1 strains and this erroneous sequence was omitted from the results. While the primer set used for this first run proved highly successful, the fraction of BPV-specific reads in some of the samples was low, indicating significant off-target amplification (Additional file 1).

To increase target-specificity, reducing the fraction of non-usable data and allowing more samples to be run on the same flow cell, a second version of the primer set was designed (V2). Because it was observed that a single sample could contain multiple complete genomes, the overall amplicon length was increased and made uniform (1400–1600 bp), and their overlapping fraction was increased slightly to facilitate genome reconstruction and phasing. The number of amplicons in this second set was reduced to eight and their specificity was limited to BPV-1 and BPV-2, allowing the degree of degeneracy to be lowered considerably. This second set was used to amplify a second batch of 24 samples. Strong PCR amplification was observed in eighteen samples, and a complete genome sequence could be retrieved for all of these samples following nanopore sequencing. The fraction of BPV-specific reads was also significantly increased compared to the first run, highlighting the higher specificity of the V2 primer set (Additional file 1). Conversely, only limited amplification (< 5 ng/µl) was observed in the remaining six samples. Therefore, these samples were initially not used for nanopore sequencing. Compared with the eighteen successful samples, these six samples all had low viral loads, as determined by qPCR. Amplification of these six samples was also reattempted using the V1 primer set, after which both the V1 and V2 amplicons were used for a nanopore run. This run failed to yield any complete genomes, indicating that failure to amplify with the V2 primer set is correlated with failure to sequence. Additionally, failure to yield complete genomes seems not linked to the primer set used, but dependent on sample quality.

Because two of the genomes from batch 2 contained a large deletion, disrupting one of the primer sites and leading to amplicon dropout, an alternative primer was designed for one of the amplicons. This slightly modified primer set (V3) was used to amplify a final batch of 30 samples. Ten samples, all with low viral loads, failed to amplify sufficiently. The remaining twenty samples were used for nanopore sequencing, each yielding a complete viral genome. However, it was observed in most samples that amplicon 2 underperformed compared to the other amplicons, requiring higher overall read counts to ensure sufficient sequencing depth.

### Genome characterization

**Fig. 1:**
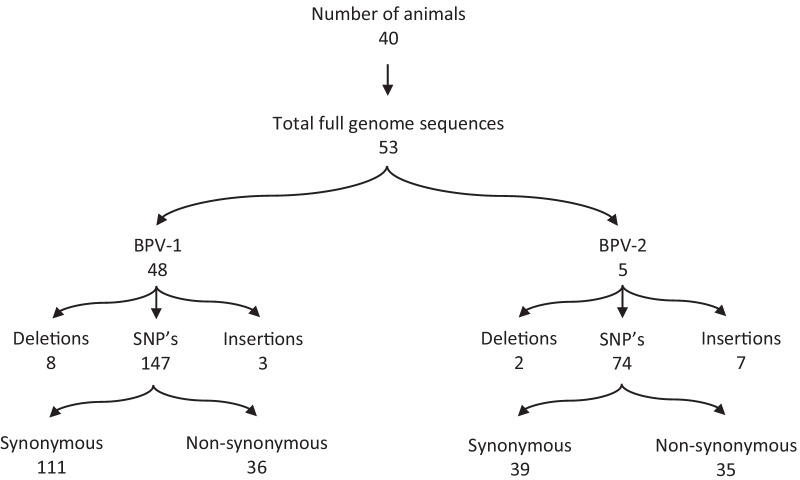
Classification and number of sequence variations. Mutations identified in BPV were classified according to their genotype. Variations were further classified based on their type of genetic mutation

Taken together, we managed to retrieve 53 complete genomes with a minimum coverage > 100x, 48 corresponding to BPV-1 and 5 to BPV-2, from 49 individual samples (Fig. 1). BPV-1 variants were highly similar to the Refseq (NC_001522) sequence, according to the observed 99.13–99.80% nucleotide identity as evaluated by BLAST. BPV-2 variants were slightly more divergent, according to the observed 98.77–98.82% similarity with the BPV-2 reference (M20219). A complete overview of the genome organization, highlighting all mutations compared to the corresponding reference sequence, can be found in Additional file 3 (BPV-1) and Additional file 4 (BPV-2). Overall, more than 120 unique substitutions were observed in the BPV-1 variants, with some being present in all our sequences. Thirty-six substitutions were non-synonymous, spread across the different genes. E2 had an especially high fraction of non-synonymous mutations, with 10/12 observed substitutions resulting in an amino acid change. Interestingly, several of the BPV-1 variants had (multiple) major deletions (up to 603 nucleotides) in the L1 and L2 genes or the LCR. In three samples (1.7, 3.20, 3.24), these deletions were only present in part of the sequencing data, and the non-deleted variant could be detected in parallel. Intriguingly, no other mutations were observed at comparable frequencies in these samples, suggesting that these deletions are somatically acquired and not indicative of a mixed infection with multiple strains.

### Phylogeny

**Fig. 2:**
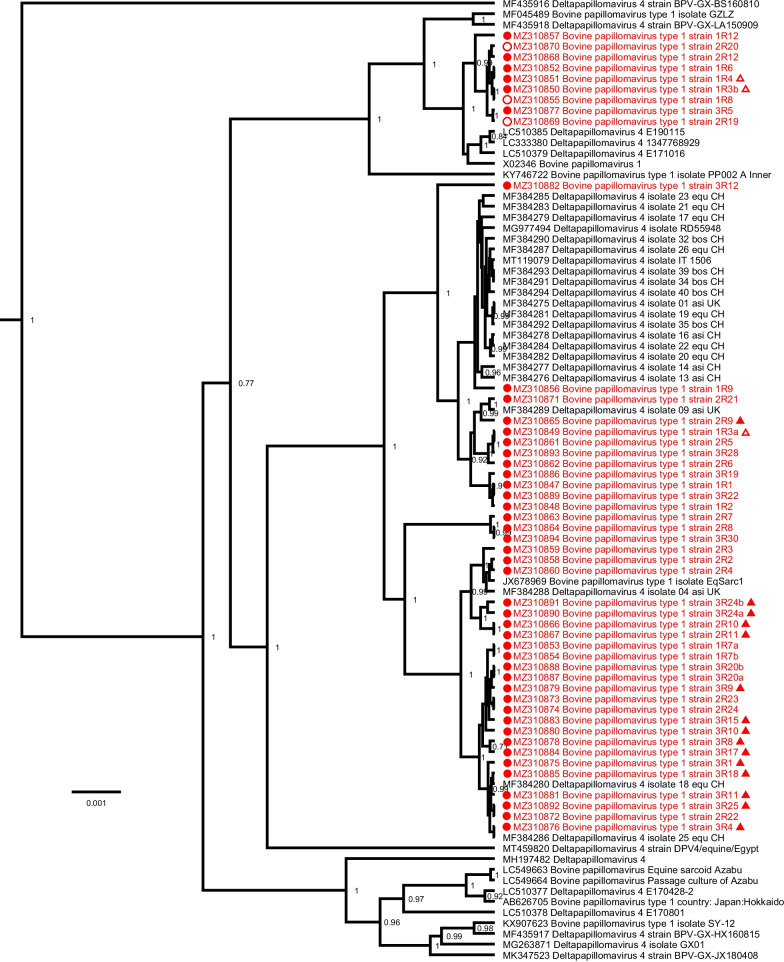
Bayesian phylogenetic tree of all currently available complete BPV-1 genomes. Sequences discovered in this study are marked in red and are preceded by a red circle. Bovine sequences are preceded by an empty circle. The origin of non-Belgian sequences are indicated by an empty or a solid triangle referring to Luxembourg or France, respectively. Branch lengths are drawn according to scale, denoting the number of nucleotide substitutions per site. Numbers at the nodes indicate posterior support values. Only values > 0.7 are shown

**Fig. 3:**
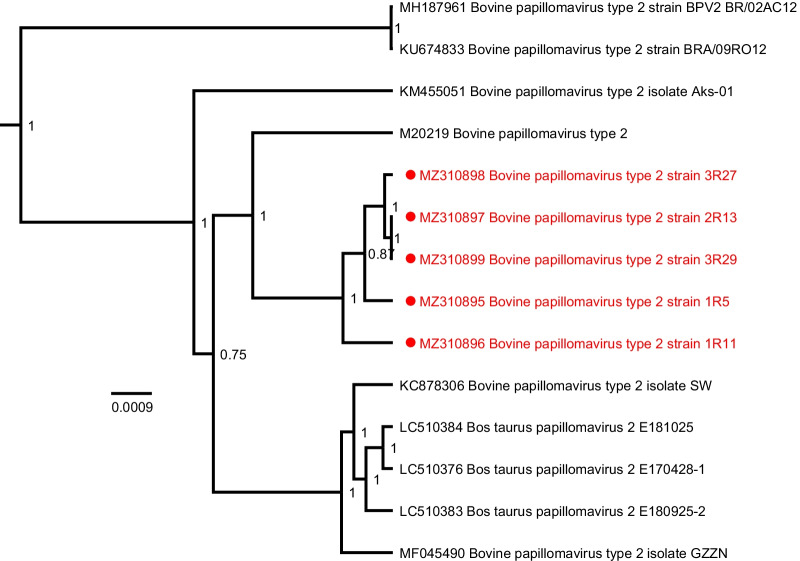
Bayesian phylogenetic tree of all currently available complete BPV-2 genomes. Sequences discovered in this study are marked in red and are preceded by a red circle. All BPV-2 sequences were isolated from Belgian horses. Branch lengths are drawn according to scale, denoting the number of nucleotide substitutions per site. Numbers at the nodes indicate posterior support values

Phylogenetic analysis of the BPV-1 sequences with all known complete-length BPV-1 references, based on the complete nucleotide sequence, shows the sequences described in this study clustering at different positions throughout the tree (Fig. 2). Mutations were found spread over the different geographical locations (Belgium, France, Luxembourg), species (equine or bovine) and clinical presentations. The five BPV-2 sequences described here are closely related to each other and form a single group clustering near the BPV-2 reference sequence M20219 (Fig. 3).

## Discussion

In Europe, sarcoids are mainly caused by BPV-1, with BPV-2 being detected in only 10% of lesions [17, 22]. The most abundant BPV type identified in the analyzed samples was type 1, confirming the main role of this type in the etiology and pathogenesis of equine sarcoids in Europe [17, 31–33]. Only five out of 53 sequences were identified as BPV-2. Due to the low sample load for this type, no conclusion could be made regarding sequence variation for sarcoid-derived BPV-2 sequences. On the other hand, by significantly expanding the number of available type 1 whole genome sequences, we managed to identify substantial genetic variation among isolated BPV-1. Whereas earlier genetic studies mainly focused on the ORF of a confined set of genes, our optimized next-generation sequencing (NGS) protocol facilitates the discovery of nucleotide changes in unexpected genetic regions, such as non-coding promotor regions. Furthermore, the use of a high-fidelity polymerase for the generation of the amplicon library minimizes the risk of observed mutations being attributable to amplification errors, although their occurrence cannot be entirely ruled out. Knowledge of sequence variation in these regions, particularly the LCR, is important because they may have an impact on the transcriptional efficiency of the virus. By introducing such progressive methodology in equine sarcoid research, a significant amount of full-length sarcoid-sourced BPV sequences could be generated. Therefore, this study provides valuable information for future research regarding the biology of cross-species BPV infection and its association with the equine sarcoid. By generating a significant amount of whole genome sequence information for a variety of clinical samples, originating from different tumour types and/or host species from three different geographical areas, our customized NGS protocol proved to be applicable for a highly diverse sample collection. Moreover, our approach of genomic characterisation allows us to generate multiple whole genomes from a clinical sample, which was not possible so far. The limited geographical area needs to be pointed out as a limitation of this study. Therefore, analysing samples from other continents could be of interest to verify if the results observed here could be extrapolated to sarcoids worldwide.

In the current study, we detected 33 type-1 substitutions in equine derived samples resulting in an amino acid change. The possible impact of these amino acid changes upon protein structure and function remains to be elucidated. A fraction of the multiple single-nucleotide polymorphisms (SNPs) was present in all the samples, in contrast to the SNPs that were only found in several samples.

BPV variation ranged from minor amino acid substitutions to notable sequence deletions. In one sample, we identified a deletion of 169 nucleotides (nt. 7435–7604) situated in the non-coding LCR region. Further variation in this region included other smaller deletions and a multitude of substitutions. Interestingly, three single non-coding nucleotide substitutions were located within different E2 binding sites (BS). While these findings are in accordance with the results of [27] that showed identical SNPs G7595T and A7598C in BS6, the SNP detected in BS8 (G7642A) differed by 2 nucleotides compared with our results (G7644A). It has been documented that several nucleotide changes within the LCR are enough to increase transcriptional activity [34]. Therefore, the identified LCR variants may have functional significance, contributing to the development of sarcoid tumours. In the different BPV genes, we see varying rates of non-synonymous mutations, with especially E2 (83%) showing a common occurrence of such mutations (Additional file 3). Intralesional expression of early BPV-1/-2 regulatory oncoproteins, including the E2 protein, supports the role of BPV in the multifactorial etiology of equine sarcoids. During the early phase of infection, E2 proteins interact with the LCR fragment in the viral genome and regulate transcription of other early and the late genes [19, 35, 36]. Since we found such a high fraction of non-synonymous E2 mutations in combination with variation in different E2 BS, further studies to explore the role of E2 variants in sarcoid pathogenesis and cross-species BPV transmission seem to be worthwhile. In this context, equine-associated BPV-1 LCR variants have previously shown functional significance in vitro due to higher transcriptional activity in equine cells, suggesting that these BPV variants have an enhanced function in the equine host [27].

Genomic studies of equine-associated BPV have been reported worldwide [19, 22, 23, 37]. However, these have been restricted almost exclusively to partial sequencing of selected genetic regions, with the LCR and E5 being the most extensively studied regions. Conversely, complete sarcoid-derived BPV genomes have rarely been sequenced, in part due to technical limitations. Sequence variation was previously detected in the ORF of E2, the LCR [27, 38] and the E5 ORF [19, 22, 23]. Interestingly, Federica et al. [23] identified both mutant and reference E5 in the same subclinically infected horses, while sarcoid-bearing horses were only infected by virus containing mutant E5 DNA. Accordingly, the vast majority (87%) of the sarcoid-sourced BPV-1 in this study contained mutations in the E5 ORF. Nevertheless, reference E5 was also present in six sarcoid samples. One of these samples (Additional file 1: batch 1, sample 3) contained multiple sequence variants: 1R3b with reference and 1R3a with mutant E5 (Additional file 3). In terms of the LCR region, one particular LCR variant (SV20) could only be found in equine samples and in none of the thirty bovine samples analyzed by Nasir et al. [27] and Trewby et al. [38]. The equine and bovine samples in our sample set showed some overlap in sequence variation. However, our bovine sample set is too limited to exclude possible host-specific variation.

The E5 nucleotide region is an important region because it codes for the major viral oncoprotein, which has transforming capacity in equine cells [24]. Regarding the E5 nucleotide region in the analyzed samples in the present study, the glutamine at residue 17 was constant in all of the variant E5 predicted protein sequences. Since the integrity of this residue is crucial for inducing cell transformation [39, 40], the function of the described E5 variants seems to be preserved. Fourteen out of 39 BPV-1 E5 variants contained the same nucleotide substitution G3920T (gene position 43). Interestingly, this substitution was found in samples from sarcoid-bearing horses in both Belgium and neighboring countries (Luxembourg and France), indicating that this variant is currently prevalent in Western Europe. The G/T substitution at position 43 leads to the change of an alanine with a serine amino acid residue. The two other substitutions T3886C (gene position 9) and A3937G (gene position 60) did not alter the deduced amino acid sequence. All of these E5 mutations were previously described as potential equine adapted BPV strains by other authors [20, 22], with the A/G substitution at position 60 being constantly identified in sarcoid-bearing horses by Federica et al. [23]. The impact of these mutations remains unclear.

Considering the overall distribution of deletions in our sample set, it is remarkable that the majority of them cluster within the region coding for late viral genes. Together with the extensiveness (up to 603 nucleotides) of the described deletions, this suggests an altered function of L1/L2 in disease pathogenesis. In the past, it was believed that late viral genes were not transcribed in sarcoid lesions. Nevertheless, Wilson et al. [19] detected L2 transcripts in cDNA samples from 6 sarcoids and L1 protein has been shown to exist in association with viral DNA in some sarcoid tissues [20]. Although late gene fragments belong to the most conserved regions among the genus *Deltapapillomavirus*, minor adjustments in their nucleotide sequence may generate a shift in protein function or conformational changes in protein structure. Hereby, external epitopes functioning as immunological binding sites may possibly become incompatible with neutralizing antibodies. This could explain the contrast between the spontaneous regression of BPV induced papillomas in the authentic, bovine host and the persistent and recurrent character of equine sarcoid lesions. After all, neutralizing antibodies are considered the main protection factor against experimental and natural infection [41]. However, the available evidence in favor of a full productive life cycle in the equine host is very limited. In this context, the identification of L1/L2 alterations of such a substantial proportion (up to 603 nucleotides) in our sample set leads us to believe that late viral gene function of these sarcoid-sourced BPV variants seems unlikely. The existence of sarcoid-derived BPV variants with loss of late viral gene function may be the result of the inability of BPV to support the vegetative portion of the viral life cycle in the horse population. Interestingly, the deletion of residue 93, 94, 95 and 414 within the L2 ORF described by Wilson et al. [19] is almost identical with the L2 deletion (nt. 4463–4472; nt. 5424–5427) present within the L2 ORF of the BPV1 sequences in our data set. This was accounted for by several extra missing bases, which caused the deletion of two extra amino acids, i.e. residue 96 (glycine) and residue 413 (tyrosine). The removal of the extra residue 96 in our samples, above the dismissal of residues 93–95, generates a novel heptamer motif (GSRATRT). These results contradict the motif GSRAGTR being a widespread motif within equine sarcoid-associated BPV, as proposed by Wilson et al. [19].

Interestingly, several of the major deletions in the BPV-1 variants were only present in part of the sequencing data, and the non-deleted variant could be detected in parallel. Concerning the detection of multiple BPV-1 variants originating from a single tumour, there are two possible scenarios: a simultaneous infection with different subtypes, or the virus can mutate at a high rate to adjust to its environment. Additional file 3 shows that no other mutations were observed at comparable frequencies in these samples, suggesting that the deletions are likely somatically acquired and not indicative of a mixed infection with multiple strains. However, it remains to be determined how this acquisition would have occurred and whether these mutants are still functional genomes or merely defective copies that nonetheless seem to be getting replicated. In the same way, our results show the presence of multiple intratypic BPV variants in sarcoids of different clinical types residing within the same horse. In contrast to HPV intratypic variants, no correlation could be observed between BPV subtype and disease severity, reflected in the clinical presentation of the equine sarcoid (Fig. 2, Additional file 1).

A previously published research paper concerning the epidemiology of sarcoids in donkeys provided supporting evidence for the concept of sarcoid transmission between equids [24, 42]. In this context, the hypothesis that allows viral spread among the horse population in the absence of an obvious bovine source is further strengthened by the identification of intratypic sequence variation in the E2-binding region of sarcoid-associated BPV [19, 22, 23, 27, 38]. Some of these earlier described variants, located within the E2-binding LCR regions, were also found in our sample set. Likewise, the entire sequence variation we detected in the E5 ORF is in compliance with the results of sarcoid-sourced samples sequenced in earlier reports. Whether the detected sequence variants are equine-adapted BPV strains able to circulate within the horse population remains uncertain. Nevertheless, the extensive somatic deletions we described in the late region of BPV-1 originating from sarcoid lesions could indicate that the second stage of transcription is not conducted in equine cells.

## Conclusion

In this study, we succeeded to introduce NGS to veterinary research focusing on the equine sarcoid, thus facilitating the first report of nanopore-based sequencing of complete BPV-1/-2 genomes originating from sarcoid lesions. Moreover, to date, this is the first description of simultaneous nanopore sequencing of multiple complete genomes originating from a single clinical sample. By implementing such state-of-the-art approach, we were able to significantly expand the number of full-length sarcoid-sourced BPV sequences. In conclusion, we found substantial genetic variation among sarcoid-derived BPV-1, although this variation could not be linked to disease severity. Nevertheless, full genome sequencing of sarcoid-associated BPV continues to be of use for elucidating the unknown mechanisms underlying other aspects of sarcoid pathogenesis, e.g. the mechanism of virus-induced cell transformation, cross-species transmission or the insufficient immune response, all of which are important regarding the central role of BPV in the multifactorial etiology of sarcoid lesions.

## Supplementary Information

**Additional file 1.** Detailed information on the clinical samples and the corresponding sequence variants. Clinical samples were processed in three different sample batches. While the primer set used for the first batch proved highly successful, the fraction of BPV-specific reads in some of the samples was low, indicating significant off-target amplification. The higher specificity of the V2 primer set significantly increased the fraction of BPV-specific reads.

**Additional file 2.** Overview of all used primer sets for the amplification of complete BPV genomes. The primer set used in batch 1 (V1) consisted of ten overlapping amplicons, divided across two pools, that spanned the complete BPV genome. These primers were degenerated at multiple positions to allow them to amplify nearly all members of the species *Deltapapillomavirus 4*, including types 1, 2 and 13. To increase target-specificity, reducing the fraction of non-usable data and allowing more samples to be run on the same flow cell, a second version of the primer set was designed (V2). The number of amplicons in this second set was reduced to eight and their specificity was limited to BPV-1 and BPV-2, allowing the degree of degeneracy to be lowered considerably. Because two of the genomes from batch 2 contained a large deletion, disrupting one of the primer sites and leading to amplicon dropout, an alternative primer was designed for one of the amplicons. This slightly modified primer set (V3) was used to amplify a final batch of 30 samples.

**Additional file 3.** A complete overview of the genome organization, highlighting all BPV-1 mutations compared to the corresponding reference sequence. Overall, more than 120 unique substitutions were observed in the BPV-1 genomes, with some being present in all our sequences. One of these samples (batch 1, sample 3) contained multiple sequence variants: 1R3b with reference and 1R3a with mutant E5. Interestingly, several major deletions in the BPV-1 genomes were only present in part of the sequencing data, and the non-deleted variant could be detected in parallel.

**Additional file 4.** A complete overview of the genome organization, highlighting all BPV-2 mutations compared to the corresponding reference sequence. We managed to retrieve 53 complete genomes, 5 corresponding to BPV-2. Due to the low sample load for this type, no conclusion could be made regarding sequence variation for sarcoid-derived BPV-2 sequences.

## Data Availability

All data generated or analyzed during this study are included in this published article and its supplementary information files.

## Abbreviations

BPV: Bovine papillomavirus
BS: Binding site
FNA: Fine-needle aspiration
NGS: Next-generation sequencing
NTC: No-template control
SNPs: Single-nucleotide polymorphisms
